# Progress Toward Poliomyelitis Eradication — Pakistan, January 2017–September 2018

**DOI:** 10.15585/mmwr.mm6744a5

**Published:** 2018-11-09

**Authors:** Christopher Hsu, Abdirahman Mahamud, Muhammad Safdar, Joanna Nikulin, Jaume Jorba, Kelley Bullard, John Agbor, Milhia Kader, Salmaan Sharif, Jamal Ahmed, Derek Ehrhardt

**Affiliations:** ^1^Global Immunization Division, Center for Global Health, CDC; ^2^World Health Organization, Islamabad, Pakistan; ^3^National Emergency Operation Center, Islamabad, Pakistan; ^4^World Health Organization, Amman, Jordan; ^5^Division of Viral Diseases, National Center for Immunization and Respiratory Diseases, CDC; ^6^IHRC, Inc, Atlanta, Georgia; ^7^United Nations Children's Fund, Islamabad, Pakistan; ^8^Department of Virology, National Institute of Health, Islamabad, Pakistan; ^9^World Health Organization, Geneva, Switzerland.

Among the three wild poliovirus (WPV) serotypes, only WPV type 1 (WPV1) has been reported in polio cases or detected from environmental surveillance globally since 2012. Pakistan remains one of only three countries worldwide (the others are Afghanistan and Nigeria) that has never had interrupted WPV1 transmission. This report documents Pakistan’s activities and progress toward polio eradication during January 2017–September 2018 and updates previous reports (*1*,*2*). In 2017, Pakistan reported eight WPV1 cases, a 60% decrease from 20 cases in 2016. As of September 18, 2018, four cases had been reported, compared with five cases at that time in 2017. Nonetheless, in 2018, WPV1 continues to be isolated regularly from environmental surveillance sites, primarily in the core reservoir areas of Karachi, Quetta, and Peshawar, signifying persistent transmission. Strategies to increase childhood immunity have included an intense schedule of supplemental immunization activities (SIAs), expanding and refining deployment of community-based vaccination implemented by community health workers recruited from the local community in reservoir areas, and strategic placement of permanent transit points where vaccination is provided to mobile populations. Interruption of WPV1 transmission will require further programmatic improvements throughout the country with a focus on specific underperforming subdistricts in reservoir areas.

## Oral Poliovirus Vaccine (OPV) Coverage and Immunization Activities

**OPV coverage.** Based on World Health Organization (WHO) and United Nations Children's Fund (UNICEF) estimates for 2017, routine vaccination coverage of infants in Pakistan with 3 doses of oral poliovirus vaccine (OPV3) by age 1 year was 75%, the same as for 2016 (*3*). Variation in OPV3 coverage among provinces is high; the highest reported administrative OPV3 coverage rates in 2017 (based on records from vaccination sites) were in Azad Jammu and Kashmir (95%) and Islamabad (91%), and the lowest were in Balochistan (35%) and the Khyber Pakhtunkhwa Tribal Districts (KP-TD) (50%).

Vaccination history (based on vaccination cards or parental recall) of children aged 6–23 months with acute flaccid paralysis (AFP) who tested negative for poliovirus (nonpolio AFP) is used as a surrogate estimate of OPV coverage in the target populations; the focus is on children who never received OPV through SIAs or routine immunization services (i.e., zero-dose children). Provinces with the highest percentage of zero-dose children were Balochistan in 2016 (2%), Gilgit-Baltistan in 2017 (15%), and Balochistan, Azad Jammu and Kashmir, Islamabad, and Gilgit-Baltistan in 2018 (each 1%).

**Supplementary immunization activities.** During January 2017–September 2018, nine nationwide SIAs and eight subnational SIAs were conducted using bivalent OPV (bOPV [types 1 and 3]) in addition to 35 small-scale SIAs in response to isolation of WPV1 from environmental surveillance and persons with AFP. Two SIA rounds using injectable inactivated poliovirus vaccine (IPV) combined with bOPV were implemented in the high-risk districts and core reservoirs, targeting 3,081,900 children in 2017 and 1,287,835 children in 2018 in Balochistan, KP-TD, and Karachi. The quality of SIAs is assessed in subdistricts (Union Councils) by post-campaign monitoring surveys, which are not random, and lot quality assurance surveys that are implemented using a random selection of clusters. Both methods have indicated high overall SIA quality, but there are some Union Councils noted to underperform frequently.

**Community-based vaccination and permanent transit points.** Locally recruited community health workers in selected districts of core reservoir areas are responsible for vaccinating children within their communities during and between SIAs through engagement with local leaders and community members. As of August 2018, a total of 18,153 community health workers have been deployed in 16 districts in KP-TD, Balochistan, and Sindh; 85% of these community health workers are women, who can more easily enter homes in these culturally and religiously conservative areas than can men. Establishment of permanent transit points is an intervention aimed at identifying and vaccinating children in mobile populations at high risk. There are currently 1,106 permanent transit points strategically placed along major domestic migration routes and at transport hubs in all provinces and at the Afghanistan official border crossings.

## Surveillance Activities

**AFP surveillance.** During January–December 2017, all provinces exceeded the target nonpolio AFP rate of two cases per 100,000 population aged <15 years and the 80% target proportion of AFP cases with collection of adequate specimens (Table). During January 2017–September 2018, the national NPAFP rate was 12.9, ranging from 11.5 to 24.1 among provinces; the percentage of AFP cases with adequate stool specimens was 89% nationally, ranging from 87% to 95% among provinces.

**TABLE:** Acute flaccid paralysis (AFP) surveillance indicators and reported cases of wild poliovirus (WPV), by province and period — Pakistan, January 2017–September 2018

Province	AFP surveillance indicators (January–December 2017)			Reported WPV cases			
	No. of AFP cases	Nonpolio AFP rate*	% with adequate specimens^†^	Jan–Jun 2017	Jul–Dec 2017	Jan–Sep 2018	Total
**Pakistan total**	**10,318**	**12.4**	**86**	**3**	**5**	**4**	**12**
Azad Jammu Kashmir	179	10.0	83	0	0	0	**0**
Gilgit-Baltistan	531	12.1	93	1	0	0	**1**
Islamabad	107	18.4	87	0	0	0	**0**
KP-TD	2,103	17.7	82	0	1	1	**2**
Punjab	4,549	10.3	87	1	0	0	**1**
Balochistan	531	13.6	84	1	2	3	**6**
Sindh	2,184	11.5	87	0	2	0	**2**
FATA	606	30.0	88	0	0	0	**0**

**Abbreviations:** FATA = Federally Administered Tribal Areas; KP-TD = Khyber Pakhtunkhwa Tribal Districts.

* Per 100,000 children aged <15 years.

^†^ Two stool specimens collected at an interval of at least 24 hours within 14 days of paralysis onset and properly shipped to the laboratory.

**Environmental surveillance.** Environmental surveillance supplements AFP surveillance through systematic, strategic sewage sampling tested for poliovirus, currently at 59 sites. Thirty-nine (66%) of these sites have been sampled monthly during 2016–2018. Although the number of WPV1 cases in Pakistan decreased during January 2016–September 2018, the number and proportion of samples from these 39 environmental surveillance sites that tested positive for WPV1 have not substantially changed: 60 (13%) in 2016, 89 (19%) in 2017, and 53 (17%) from January–August 2018, primarily in Karachi, the Quetta block (Pishin, Killa Abdullah, and Quetta districts), and Peshawar.

## Epidemiology of WPV1 Cases

**FIGURE 1:**
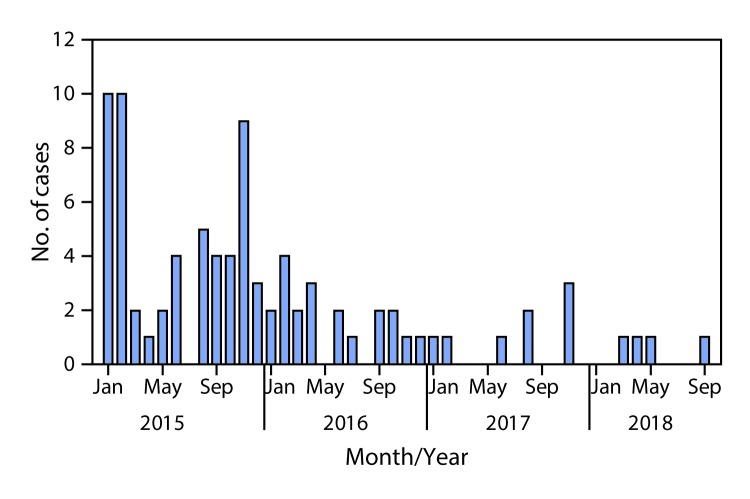
Number of cases of wild poliovirus type 1, by month — Pakistan, January 2015–September 2018

**FIGURE 2:**
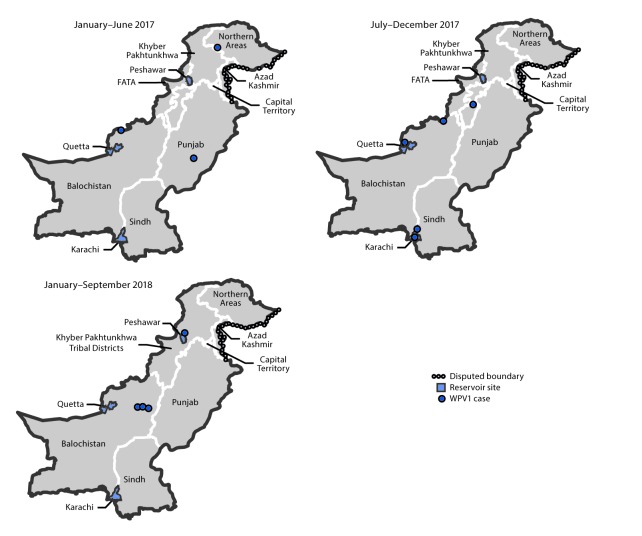
Location of cases of wild poliovirus type 1 (WPV1), by province and period — Pakistan, January 2017–September 2018 **Abbreviation:** FATA = Federally Administered Tribal Areas.

During 2017, eight WPV1 cases were reported in Pakistan, a 60% decrease from the 20 cases reported in 2016 (Figure 1). Four WPV1 cases have been reported during January–September 2018 in two districts (Dukki in Balochistan and Charsada in KP-TD), compared with five WPV1 cases during the same period in 2017 in five districts. Of the eight WPV1 cases reported in 2017, one was reported from each of three provinces (KP-TD, Gilgit Baltistan, and Punjab), two from Sindh, and three from Balochistan (Figure 2). Of the 20 WPV1 cases reported in 2016, eight (40%) were from KP-TD, eight (40%) from Sindh, two (10%) from Federally Administered Tribal Areas, and two (10%) from Balochistan. The ages of the 12 children with WPV1 cases reported during January 2017–September 2018 ranged from 4 to 38 months. Based on parental recall, none of the 2018 cases occurred in zero-dose children; all had received OPV during SIAs (from 3 to >7 doses), and one had received three OPV doses and one IPV dose through routine immunization services. During the same period in 2017, one child with WPV1 (20%) had received zero doses, one (20%) child had received only SIA doses, and three children (60%) received doses from routine immunization services and during SIAs.

## Discussion

Maintaining intensively scheduled SIAs with high overall quality indicators has been associated with the trend of decreasing WPV1 cases in Pakistan. At the same time, AFP surveillance and environmental surveillance sampling indicate the persistence of WPV1 transmission in 2018 in three key reservoirs. WPV1 transmission in those reservoirs will not be interrupted without fully addressing low SIA quality and weak routine immunization services in specific Union Councils.

Despite advances over previous years, parental refusals of OPV vaccination have increased and pose a substantial challenge to reaching all children in core WPV reservoir areas (*4*). To address this growing trend, an aggressive communication strategy has been implemented through traditional and social media as well as targeted engagement with communities and their opinion leaders. The main factors contributing to mounting refusals have been the recent rapid spread of misconceptions about overall vaccine safety and efficacy and demand for basic services other than polio vaccination (e.g., clean water, maternal health services, and adult health needs) in marginalized urban communities. Frontline vaccinators are the key to reaching caregivers in households, and with enhanced communications messages, they can better counter these increasing vaccine refusals. Polio eradication partners are working with development agencies to address the demand for basic services in critical areas within the reservoirs.

Mass cross-border population movements from Afghanistan and internal migrant populations within Pakistan pose a challenge to vaccinating children. Although progress has been made in identifying and tracking mobile populations at high risk and vaccinating children at permanent transit points on major routes, these strategies can be further enhanced to overcome current challenges (e.g., refusals and failure to address travel in all directions by some teams).

In 2018, as of September 18, a total of 13 WPV1 cases have been reported in neighboring Afghanistan. The geographic locations of these cases are in two major cross-border migration areas between Afghanistan and Pakistan, forming corridors into each country: the Northern Corridor includes KP-TD Province in Pakistan, and the Southern Corridor includes Balochistan (*5*). Genetic sequencing data from environmental isolates indicate that the WPV1 found in Pakistan has also been detected in neighboring provinces in Afghanistan. Genomic sequence analysis of WPV1 isolated from patients with AFP has also shown linked cross-border transmission. For several years, the two countries have been fully synchronizing SIAs and conducting regular bilateral meetings through respective national and provincial Emergency Operation Centers to share data on migrant movements; however, efforts to improve bilateral coordination must be further pursued to ensure optimal vaccination of migrant populations.

Ending WPV1 transmission in Pakistan will require continuing overall high-quality SIAs and improving routine immunization services. It will also require assessing and augmenting supplemental and routine vaccination activities in poor-performing Union Councils in each reservoir.

**Summary**

**What is already known about this topic?**

Pakistan remains one of three countries (along with Afghanistan and Nigeria) where wild poliovirus type 1 (WPV1) transmission has never been interrupted.

**What is added by this report?**

During January 2017−September 2018, WPV1 cases in Pakistan continued to decrease compared with previous periods. However, environmental surveillance continues to detect polioviruses, an indication of children who were missed for immunization, as well as poor vaccination program performance.

**What are the implications for public health practice?**

Stopping WPV1 transmission will require further enhancing the quality of vaccination and surveillance, augmenting cross-border coordination with Afghanistan, strengthening efforts to reach mobile populations at high risk, and focusing on poor-performing areas.
